# Quantum control of spin-nematic squeezing in a dipolar spin-1 condensate

**DOI:** 10.1038/srep43159

**Published:** 2017-02-24

**Authors:** Yixiao Huang, Heng-Na Xiong, Yang Yang, Zheng-Da Hu, Zhengjun Xi

**Affiliations:** 1 School of Science, Zhejiang University of Science and Technology, Hangzhou, Zhejiang, 310023, China; 2 College of Computer Science, Shaanxi Normal University, Xi’an 710062, China; 3 Department of Applied Physics, Zhejiang University of Technology, Hangzhou 310023, China; 4 Jiangsu Provincial Research Center of Light Industrial Optoelectronic Engineering and Technology, School of Science, Jiangnan University, Wuxi 214122, China

## Abstract

Versatile controllability of interactions and magnetic field in ultracold atomic gases ha now reached an era where spin mixing dynamics and spin-nematic squeezing can be studied. Recent experiments have realized spin-nematic squeezed vacuum and dynamic stabilization following a quench through a quantum phase transition. Here we propose a scheme for storage of maximal spin-nematic squeezing, with its squeezing angle maintained in a fixed direction, in a dipolar spin-1 condensate by applying a microwave pulse at a time that maximal squeezing occurs. The dynamic stabilization of the system is achieved by manipulating the external periodic microwave pulses. The stability diagram for the range of pulse periods and phase shifts that stabilize the dynamics is numerical simulated and agrees with a stability analysis. Moreover, the stability range coincides well with the spin-nematic vacuum squeezed region which indicates that the spin-nematic squeezed vacuum will never disappear as long as the spin dynamics are stabilized.

The study of spin squeezing^1^,^2^,^3^ has stimulated, both theoretically and experimentally, much recent interest because of their applications in quantum physics and quantum information processing^4^,^5^. Spin squeezing is valuable resource of quantum correlations and can be used to detect quantum entanglement^6^,^7^,^8^,^9^,^10^. Beside its intrinsically fascination, squeezing is demonstrated as one of the most tested schemes in precision measurement to go beyond the standard quantum limit (SQL)^11^,^12^. Since then, many efforts have been devoted to the generation of squeezing in atomic systems, such as generating spin squeezing in atomic ensembles via atom–photon interactions^13^,^14^,^15^,^16^,^17^,^18^,^19^, and in Bose-Einstein condensates (BECs) via atomic collisions^20^,^21^.

The atomic squeezed states which are introduced in contrast to coherent spin states, were first considered for a system of two-level atoms. For spin-1/2 particles, the state can be uniquely specified by different components of the total spin vector **S** = (*S*_*x*_, *S*_*y*_, *S*_*z*_). For the spinor-1 atomic BECs^22^,^23^,^24^,^25^,^26^,^27^,^28^,^29^,^30^,^31^,^32^,^33^, a natural basis to describe the wavefunction can be specified in terms of nematic tensor *Q*_*i,j*_({*i, j*} ∈ {*x, y, z*})^34^,^35^,^36^,^37^,^38^,^39^,^40^ in addition to the usual spin vector **S**. In matrix form, *Q*_*i,j*_ can be written as *Q*_*i,j*_ = *S*_*i*_*S*_*j*_ + *S*_*j*_*S*_*i*_ − (4/3)*δ*_*ij*_ with *δ*_*ij*_ being the kronecker delta. The nematic moments *Q*_*i,j*_ and the spin vector **S** constitute SU(3) Lie algebra which suggests newtrade-off relations between the spin operator **S** and the nematic tensor *Q*_*i,j*_. It indicates that not only quantum fluctuations of the spin vector, but also those of the nematic tensor can be controlled by manipulating various types of correlations between noncommutative spin and nematic-tensor observables. Thus the squeezing can be induced by other types of correlations such as spin-nematic and internematic correlations.

Recently, spin-nematic squeezed vacuum was measured experimentally, which improved on the SQL by up to 8–10 dB^41^. Such a squeezing associated with negligible occupation of the squeezed modes, which is analogous to optical two-mode vacuum squeezing and widely application in light^42^,^43^,^44^,^45^. The dynamics stabilization was also performed in a spinor BEC by manipulating the external periodic microwave pulses, by which the atoms are always condensed in one spin component^23^. The above experiments considered the system with magnetic field and neglected the effect of the dipolar interaction. It is well known that the dipolar interaction in spinor alkali condensates may play a more prominent role in the squeezing and dynamical stability^46^,^47^,^48^,^49^,^50^. In addition, for the quantum information, beyond the generation of the squeezing itself, it is desirable to maintain the squeezing and also its direction for a long time^51^,^52^.

In this paper, we propose a scheme for storage of the maximal spin-nematic squeezing in a dipolar spinor condensate. We consider a system of dipolar spin-1 BEC with an initial state of all atoms in the state of *m*_*f*_ = 0. The free dynamical process gives rise to quantum spin mixing and spin-nematic squeezing. By manipulating an external microwave pulse at a time that maximal spin-nematic squeezing occurs, the squeezing is stored for a long time with its squeezing angle maintained in a fixed direction. The dynamic stabilization of the system is demonstrated by applying periodic microwave pulses. The range of pulse periods and phase shifts with which the condensate can be stabilized is numerical calculated and compares well with a linear stability analysis in the mean field approximation. We also show that the existence range of the spin-nematic squeezed vacuum coincides well with the stabilization range, which indicates that the spin-nematic squeezed vacuum will always exist as long as the system is stabilized.

## Results

### Model

We consider a spin *F* = 1 condensate with *N* atoms trapped in an axially symmetric potential. For simplicity, we choose the symmetry axis to be the quantization axis *z*. The second quantized Hamiltonian of the system with short-range collisions and long-range magnetic dipolar interaction reads^31^


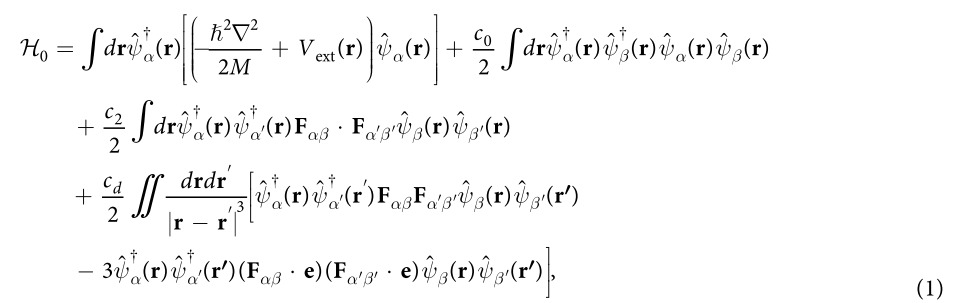


where 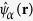 is the atomic field annihilation operator associated with atom in spin state 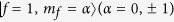, **F** is the angular momentum operator and ***e*** = (*r* − *r*^′^)/|*r* − *r*^′^| is the unit vector. The mass of the atom is given by *M* and the trapping potential *V*_ext_(**r**) is assumed to be spin independent. Collisional interaction parameters for the spin-independent and spin-exchange are 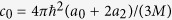 and 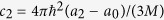, respectively^24^,^25^, where *a*_*f*_ (*f* = 0, 2) is the *s*-wave scattering length for spin-1 atoms in the combined symmetric channel of total spin *f*. The strength of the magnetic dipole-dipole interaction is given by 
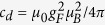
 with *μ*_0_ being the vacuum magnetic permeability, *μ*_*B*_ the Bohr magneton, and *g*_*F*_ the Landé *g*-factor. For both the ^87^Rb and ^23^Na atoms, one has 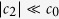 and 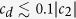. Under these conditions, the single mode approximation (SMA) is expected to be valid, and then the field operators can be decomposed as 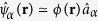
30,31, where 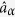 is the annihilation operator of spin component *α*. The Hamiltonian of the system under the SMA (with constant terms dropped) can be remarkably reduced to^31^


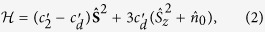


where 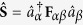 is the total angular momentum operator, 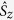 is its *z*-component, and 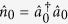. 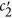 and 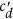 are the rescaled collisional and dipolar interaction strengths, respectively, which are given by 
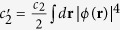
, 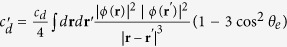 with *θ*_*e*_ being the polar angle of (**r** − **r**^′^). The sign of 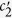 is determined by the type of atoms: ^87^Rb (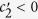) and ^23^Na (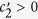), the sign and the magnitude of dipolar interaction strength 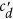 can be tuned via modifying the trapping geometry (see Methods).

### Spin-nematic squeezing

Before discussing the dynamic properties of the system, we want to point out that the term 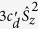 commutes with all the other terms in the Hamiltonian. If we start with an initial state that is an eigenstate of 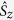, the dipolar term 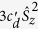 has no effect and thus can be neglected. In the following, we consider an initially spin-polarized condensate where all atoms are prepared in the spin-0 state, i.e., 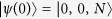, where 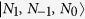 denotes the usual Fock states. During the spin-mixing dynamical processing, the spin mixing Hamiltonian (2) conserves both the total particle number *N* and magnetization, the evolution state of the system in vector form is


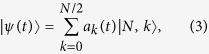


where 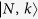 is so-called pairs basis with *N* the total particle number and *k* the number of pairs of atom in the *m*_*f*_ = ±1 states. Thus the expected values of 〈*S*_*x,y,z*_(*t*)〉 equals to zero and then the mean spin vanishes and the spin squeezing parameter is divergent.

**Figure 1:**
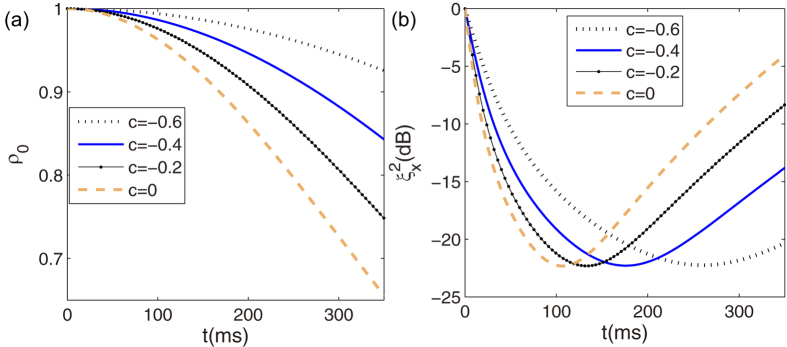
Spin population and spin nematic squeezing. (**a**) The spin component *m*_*f*_ = 0 and (**b**) the corresponding spin-nematic squeezing parameter 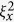 as a function of *t* with *N* = 3000.

Fortunately, spin-1 has other higher order spin moments which could exhibit squeezing. Based on the commutation relationship of the operators *Q*_*i,j*_, we can define {*S*_*x*_, *Q*_*yz*_, *Q*_+_} and {*S*_*y*_, *Q*_*xz*_, *Q*_−_} as two subspaces of SU(3), where *Q*_+_ and *Q*_−_ are defined as *Q*_+_ = *Q*_*zz*_ − *Q*_*yy*_ and *Q*_−_ = *Q*_*xx*_ − *Q*_*zz*_, respectively (see Methods). According to the generalized Heisenberg uncertainty relation 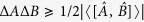, only operator pairs with non-zero expectation values for their commutation relations can exhibit squeezing. For the initial state 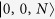, only two of the SU(3) commutators have non-zero expectation values, i.e., 〈*Q*_±_〉 ≠ 0. Thus we can obtain a uncertainty relationship between a spin operator and a quadrupole nematic operator, i.e, 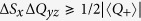 and 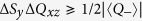. From these relations, two spin-nematic squeezing parameters can be defined in terms of quadratures of the operators^41^


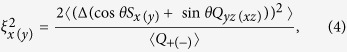


where *θ* is the quadrature angle. Consider the evolution state of the system with negligible populations of the *m*_*f*_ = ±1 states, the expectation values for two of the SU(3) commutators are given by 〈[*S*_*x*_,*Q*_*yz*_]〉 = −2*iN* and 〈[*S*_*y*_, *Q*_*xz*_]〉 = 2*iN*. In such a case, the relevant uncertainty relations between quadrupole nematic operators and spin operators are given by 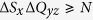 and 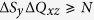. Then the squeezing parameter 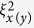 are the ratio between the variance of the quadrature operator to the standard quantum limit of *N* which reduce to^41^


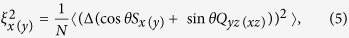


and 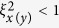 indicates spin-nematic squeezed vacuum. In Fig. 1, we display the dynamics of the spin component *m*_*f*_ = 0 (*ρ*_0_ = *N*_0_/*N*) and the corresponding spin-nematic squeezing parameter (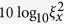) for different dipolar interactions. The spinor interaction strength is chosen as a realistic experimental parameter with 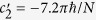 Hz, and *c* is defined as 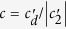. As the dipolar interaction |*c*| increases, the speed of spin mixing slows down and the corresponding time of maximal squeezing *t*_*m*_ becomes larger. It is due to the fact that the enhancement of dipolar interaction suppresses the spinor interaction. When the inter-spin interaction reduces to 0, there will be no spin mixing and squeezing.

In the recent experiment, the spin-nematic squeezing is measured by using an SU(3) rotation in spin-nematic phase space around the −*Q*_*zz*_ axis^41^. The wave function after the rotation is given by


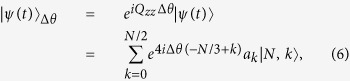


which corresponds to an additional phase on different states 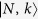. The rotation (phase shifts) can be experimentally implemented by using 2*π* Rabi pluses on the 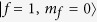 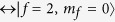 microwave clock transition, which can effectively shift the phase of the state 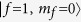 with an amount 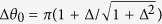, where Δ is the detuning normalized to the on-resonance Rabi rate^41^.

**Figure 2:**
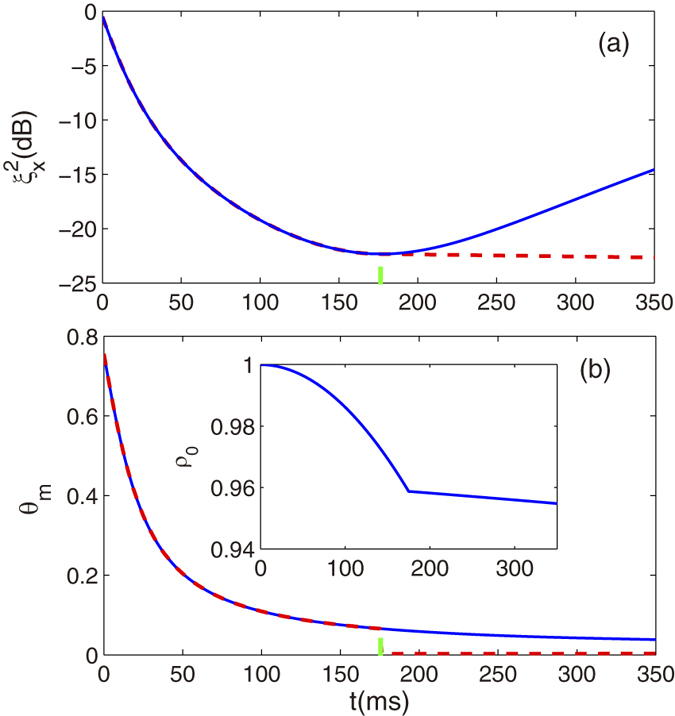
Spin nematic squeezing and squeezing angle. Time evolution of (**a**) the squeezing parameter, (**b**) the squeezing angle for the dipolar interaction *c* = −0.1 with *N* = 3000. Solid curves: the free evolution case; dashed curves: the case for pulse at the time *t*_*m*_ indicated by the colored ticks along the horizontal axis.

The microwave pulse can also be used to control the dynamics of spin-nematic squeezing. As shown in Fig. 2, a pulse is added at the maximal-squeezing time *t*_*m*_ with the phase shift Δ*θ* = −0.98*π*, we can find that the maximal squeezing can be stored for a long time. In addition, with the help of the pulse, the direction of the squeezing can also be maintained along a fixed axis. Experimentally, it is possible that the parameter *c* may deviate from the value of *c* = −0.1. We varied the dipolar interaction parameter *c* near the value of *c* = −0.1, and found that the spin-nematic squeezing (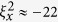
dB) can also be maintained for a long time (≈150 ms) with many other sizes of the microwave pulse parameters, such as *δθ* = −0.98*π, c* = −0.09 and *δθ* = −0.98*π, c* = −0.11. In this way, the storage of spin-nematic squeezing and the direction of the squeezing are realized by applying external microwave pulse. Here, we emphasize that the maintained squeezing is not a squeezed vacuum; as shown in the inset of Fig. 2(b), the population in spin components *m*_*f*_ = ±1 are macroscopically populated.

### Dynamic stabilization and spin-nematic squeezed vacuum

**Figure 3:**
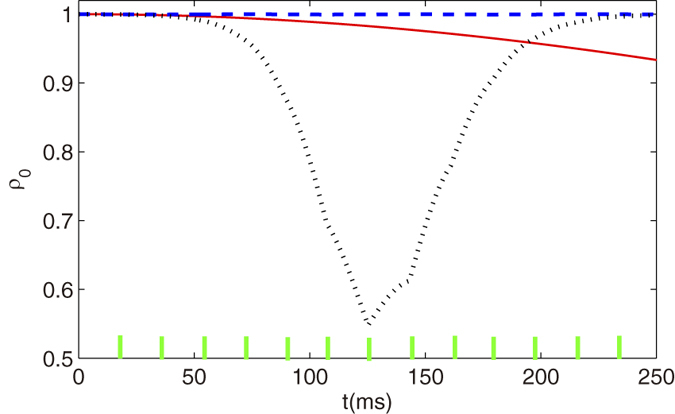
Population of spin component *m*_*f*_=0. Numerical result of *ρ*_0_ for different applied phase shifts Δ*θ* = −0.5*π* (blue dashed line),0 (red solid line), and −0.2*π* (black dot line) for stability, free and unstable cases, respectively. The ticks represent the pulses. The total particle number *N* = 3000 and the phase period is 18ms with the first pulse applied at 18ms.

Next we consider a spin-nematic squeezed vacuum which is associated with negligible occupation of the squeezed modes, and is analogous to optical two-mode vacuum squeezing^42^,^43^,^44^,^45^. To generate the spin-nematic squeezed vacuum, we shall control the stability of the dynamics which ensure that there is essentially no population transfer (<1%) from the *m*_*f*_ = 0 state. In our scheme, the dynamic stabilization is achieved by preventing the buildup of the correlations using the periodic phase shifts which is similar to that used in spin-1 condensate with quadratic Zeeman energies^23^. The numerical simulation result demonstration dynamic stabilization of the system are shown in Fig. 3. The spin population *ρ*_0_ as a function of *t* is shown for two different microwave pulse parameters with *δθ* = −0.5*π* and −0.2*π*, which respectively corresponds to a stabilized condition and a unstable condition. For comparison, the unstabilized dynamics showing free evolution spin mixing with *δθ* = 0 is also displayed. The difference between the three different cases is the size of quadrature phase shift applied per pulse. It means that for a proper size of quadrature phase shift, the dynamic of the system can be stabilized and then measurement of the spin population *ρ*_0_ corresponds to a measurement of the projection of the spin nematic sphere on the polar axis. Conceptually, the spin-nematic phase space can be represented on a unit sphere with axes {*S*_⊥_, *Q*_⊥_, *x*}, where 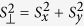, 
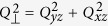
, and *x* = 2*ρ*_0_ − 1 (see Methods).

**Figure 4:**
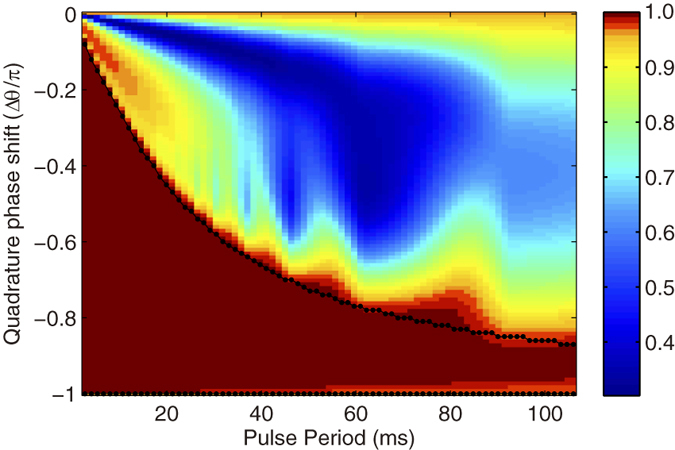
Stability range. Map of the numerical simulation of stability region for *ρ*_0_ population after 160 ms of evolution. The solid curves with dots are the analytic stability solution.

We have also investigate the range of pulse periods and quadrature phase shifts which provide stabilization of the spin dynamics. The numerical result of the spin population *ρ*_0_ after 160ms is also shown in Fig. 4, which displays a map of the stability region versus pulse period and quadrature phase shift. For the shorter pulse periods, the system is stabilized with a wider range of quadrature phase shifts. For long pulse periods, the range of quadrature phase shifts capable of stabilizing the dynamics shrinks. Here we also note that the direction of the shrink only along quadrature phase shifts from 0 to −*π*.

The nature of the stability can be well understood in the classical spin-nematic phase space. In the mean field framework, the evolution dynamics of *S*_⊥_ and *Q*_⊥_ are given by (see Methods)


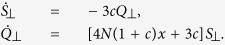


With the period quadrature phase shift 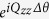, the stabilization condition of the dynamics is given by the inequality


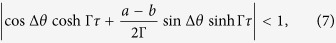


where 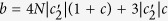, 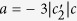 and 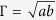. Such an inequality can be used to mark the boundaries of the analytic stability region. In Fig. 4, the analytical results of the range of the stabilization are plotted as black lines with dots in the plane of quadrature phase shifts and pulse periods. It is clearly seen that the numerical results coincide well with the analytical ones obtained with mean field approximation. Here we emphasize that the result of Eq. (7) is similar with that obtained in spin-1 condensate with external magnetic field^23^.

**Figure 5:**
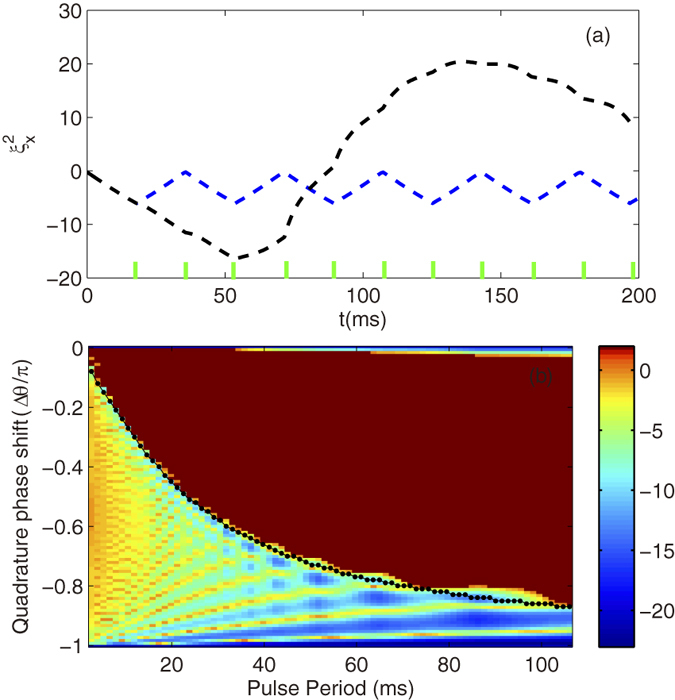
Spin nematic vacuum squeezing and squeezing range. (**a**) Evolution of spin-nematic squeezing parameter 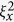 for two different quadrature phase shifts *θ* = −0.7*π* (blue dashed line) and *θ* = −0.3*π* (black dotted line). The phase period is 18 ms and the ticks represent the pulses. (**b**) Map of the numerical result for 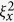 after 160 ms of evolution. The black line with dots is the stable boundary obtained by Eq. (7) in mean field approach.

When the condensate is stabilized with *ρ*_0_ = 1, the squeezing parameter 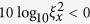
dB indicates the condensate exhibits spin-nematic squeezed vacuum. In Fig. 5(a), the evolution of the spin-nematic squeezed vacuum parameter are plotted for two different applied phase shifters. In the unstabilize case with Δ*θ* = −0.3*π*, the squeezing phenomenon disappears after a certain time. While the stabilized pulse (Δ*θ* = −0.8*π*) shows the expected periodic evolution of the spin-nematic squeezing and also show a dramatic reduction of the squeezing compared with the unstabilized one after a long time evolution. It can be noted that the system always exhibits spin-nematic vacuum squeezing with the stabilized pulse.

We also explore the range of pulse periods and quadrature phase shifts that provide the exhibition of spin-nematic squeezed vacuum for any time. The numerical results of 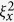 after 160 ms are shown in Fig. 5(b) which displays a map of the squeezed vacuum region versus pulse period and quadrature phase shift. For clearly shown in Figure, we set 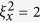 dB when 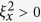 dB, which denotes no squeezing. The numerical results are in good overall agreement with the stabilization condition, which indicates that the system can always exhibits spin-nematic squeezed vacuum as the spin dynamics is stabilized. We shall point out that the the squeezing region includes unstable pulse with Δ*θ* = 0 and *π*, since the squeezing parameter 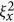 has not enough time to increase larger than 0 in 160ms for the marginally unstable case.

## Discussion

In this article,we have investigated the coherent control spin-nematic squeezing and dynamic stabilization in a spin-1 condensate with dipolar interaction by periodically manipulating the phases of the states. By applying a microwave pulses at the time when the maximal spin-nematic squeezing occurs, the maximal squeezing can be stored with its squeezing angle maintained in a fixed axis. The dynamic stabilization of system is also demonstrated by the pulse. The stability diagram for the range of pulse period and phase shifts that stabilize the spin dynamics are numerical simulated and coincide well with a stability analysis in a mean field approximation. We further study the spin-nematic squeezed vacuum of the system and map the squeezing parameter region on the plane of pulse period and quadrature phase shift. The system always exhibits spin-nematic squeezed vacuum as the spin dynamics is stabilized.

Our scheme presented above demonstrate for the storage of spin-nematic squeezing and dynamical stabilization of the spin dynamics are quite robust for a wide range of parameters. Although the stabilization is demonstrated with a condensate in SMA for which the spatial dynamics are factored out, our scheme should be applicable to the control of the coupled spin or spatial dynamics that lead to domain formation in larger condensates. We hope our scheme will be realized in future experiment and also can be used to explore the quantum control of spin dynamics in other spin systems.

## Methods

### Dipole-dipole interaction

**Figure 6:**
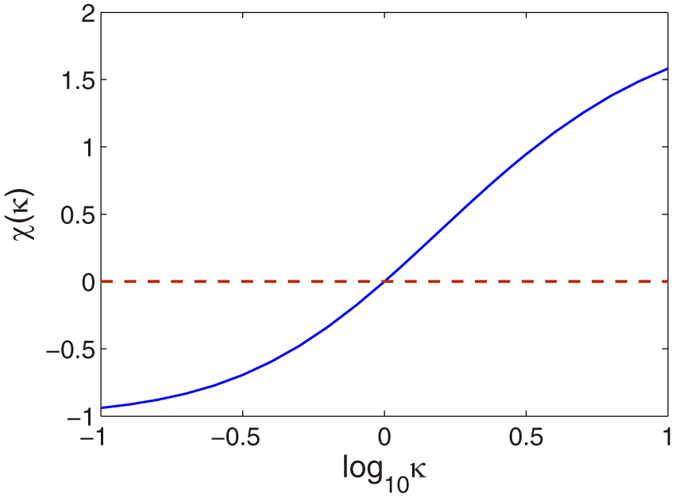
Dipole-dipole interaction. The parameter *χ(κ*) as a function of *κ*.

To calculate the parameters 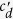 and 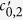, we consider *ϕ*(**r**) to be the single-particle ground state of the harmonic potential, i.e., 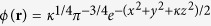, and then we can obtain


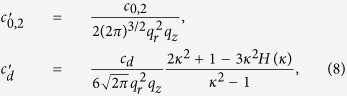


where *κ* = *q*_*r*_/*q*_*z*_ is the condensate aspect ratio and 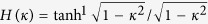. Therefore we can get 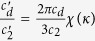 with 
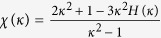
. Figure 6 shows the *κ* dependence of function *χ(κ*). It is seen that the value of the parameter *χ(κ*) can be tuned from −1 to 2 by changing the trapping geometry^31^,^32^,^33^. For *κ* < 1, the dipolar interaction is attractive, and it is repulsive for *κ* > 1. When *κ* = 1, we can obtain *χ(κ*) = 0, which indicates that the dipolar interaction disappears.

### Spin and nematic operators

According to the definition of the operator *Q*_*i,j*_ which are given by


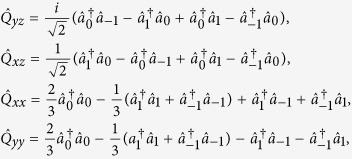


we define two SU(2) subspaces: {*S*_*x*_, *Q*_*yz*_, *Q*_*zz*_−*Q*_*yy*_}, {*S*_*y*_, *Q*_*xz*_, *Q*_*xx*_−*Q*_*zz*_}. The detail commutation relationship for these two subspaces is shown in Table 1. In fact, the spin-nematic squeezing is identical in these two subspaces.

### Classical spin-nematic phase space

Under the SMA, the spin part of the wave function can be represented by a complex vector 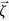 = (*ζ*_1_, *ζ*_0_, *ζ*_−1_)^*T*^ where *ζ*_*i*_ = 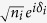 denotes the amplitude and phase of the *i*th component. For the initial condition with the fractional magnetization *m* = *ρ*_1_−*ρ*_−1_ = 0, which is a constant of the motion, a convenient parameterization of the vector *ζ*_*i*_ is given by


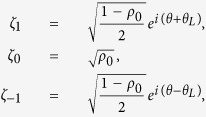


where *θ*_*L*_ = (*θ*_1_ − *θ*_−1_)/2 is the Larmor recession phase, and *θ* = (*θ*_1_ + *θ*_−1_ − 2*θ*_0_)/2 is the quadrature phase. Corresponding, the mean field expectation of the operators can be expressed as


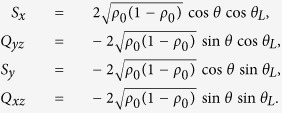


Defining 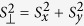, 
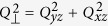
, and *x* = 2*ρ*_0_ − 1, we obtain


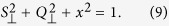


We note that *S*_⊥_, *Q*_⊥_, and *x* have spin Poisson brackets and thus define as a spin representation which can be shown as a sphere.

### Stabilization Condition

The free spin mixing dynamics in a dipolar interaction is described by


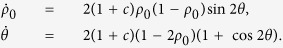


Then the evolution dynamics of *S*_⊥_ and *Q*_⊥_ are given by


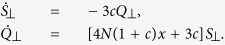


To discuss the problem of the dynamical stability, we shall adopt the linear stability analysis that has wide applications in various nonlinear systems. First, the infinitesimal variables *δS*_⊥_ and *δQ*_⊥_ are introduced by 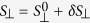, 
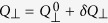
, and *x* = *x*^0^ + *δx*, where 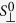, 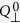 and *x*^0^ denote the expectation for the initial state which are given by 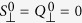, *x*^0^ = 1. Keeping the linear terms and eliminating the higher order terms, the linearized equations of motion are derived as


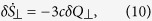



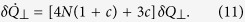


Since the expectation value of *S*_⊥_ = *Q*_⊥_ = 0 for the dynamical process, we will drop the notation *δ* of the expansion, and then Eqs (10) and (11) reduce to a matrix form


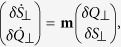


where 
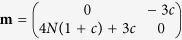
. In the plane {*S*_⊥_, *Q*_⊥_}, the quadrature phase shift corresponds to a two dimensional plane rotation matrix 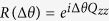 with an rotation angle Δ*θ*. Thus the full dynamics form one pulse to another including the quadrature phase shift is given by


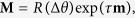


where *τ* is the pulse period. The term 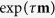 in the matrix can be written as


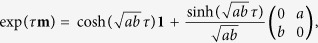


where 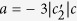 and 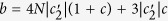. Using the same stability analysis technique employed in optical resonator theory, the dynamics of *S*_⊥_ and *Q*_⊥_ stay bounded when the trace of evolution matrix satisfies the condition |Tr[**M**]| < 2. We obtain the inequality


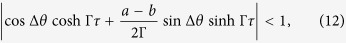


where 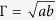.

## Additional Information

**How to cite this article:** Huang, Y. *et al*. Quantum control of spin-nematic squeezing in a dipolar spin-1 condensate. *Sci. Rep.* **7**, 43159; doi: 10.1038/srep43159 (2017).

**Publisher's note:** Springer Nature remains neutral with regard to jurisdictional claims in published maps and institutional affiliations.

## Figures and Tables

**Table 1:** Commutation relationship of the two subspaces {*S*_*x*_, *Q*_*yz*_, *Q*_+_} and {*S*_*y*_, *Q*_*xz*_, *Q*_−_}.

{*S*_*x*_, *Q*_*yz*_, *Q*_+_}	*Q*_*yz*_	*Q*_+_	{*S*_*y*_, *Q*_*xz*_, *Q*_−_}	*Q*_*xz*_	*Q*_−_
*S*_*x*_	*iQ*_+_	−2*iQ*_*yz*_	*S*_*y*_	*iQ*_−_	−2*iQ*_*xz*_
*Q*_*yz*_		2*iS*_*x*_	*Q*_*xz*_		2*iS*_*y*_

## References

[b1] Kitagawa M. & Ueda M. Squeezed spin states. Phys. Rev. A 47, 5138–5143 (1993). 9909547 10.1103/physreva.47.5138

[b2] Wineland D. J. . Spin squeezing and reduced quantum noise in spectroscopy. Phys. Rev. A 46, R6797–R6800 (1992). 9908086 10.1103/physreva.46.r6797

[b3] Wineland D. J., Bollinger J. J., Itano W. M. & Heinzen D. J. Squeezed atomic states and projection noise in spectroscopy. Phys. Rev. A 50, 67–88 (1994). 9910869 10.1103/physreva.50.67

[b4] Nielsen M. A. & Chuang I. L. Quantum Computation and Quantum Information. Cambridge University Press, Cambridge, England, (2000).

[b5] Stolze J. & Suter D. Quantum Computing: A Short Course from Theory to Experiment. second ed., Wiley-VCH Verlag, Weinheim, (2008).

[b6] Guehne O. & Tóth G. Entanglement detection. Phys. Rep. 474, 1 (2009).

[b7] Amico L., Fazio R., Osterloh A. & Vedral V. Entanglement in many-body systems. Rev. Mod. Phys. 80, 517–576 (2008).

[b8] Horodecki R., Horodecki P., Horodecki M. & Horodecki K. Quantum entanglement. Rev. Mod. Phys. 81, 865–942 (2009).

[b9] Pezzé L. & Smerzi A. Entanglement, Nonlinear Dynamics, and the Heisenberg Limit. Phys. Rev. Lett. 102, 100401 (2009). 19392092 10.1103/PhysRevLett.102.100401

[b10] He Q. Y. . Einstein-Podolsky-Rosen Entanglement Strategies in Two-Well Bose-Einstein Condensates. Phys. Rev. Lett. 106, 120405 (2011). 21517288 10.1103/PhysRevLett.106.120405

[b11] Cronin A. D., Schmiedmayer J. & Pritchard D. E. Optics and interferometry with atoms and molecules. Rev. Mod. Phys. 81, 1051–1129 (2009).

[b12] Polzik E. S. Quantum physics—the squeeze goes on. Nature 453, 45–46 (2008). 18451849 10.1038/453045a

[b13] Hammerer K., Sørensen A. S. & Polzik E. S. Quantum interface between light and atomic ensembles. Rev. Mod. Phys. 82, 1041–1093 (2010).

[b14] Estève J., Gross C., Weller A., Giovanazzi S. & Oberthaler M. K. Squeezing and entanglement in a Bose¨CEinstein condensate. Nature (London) 455, 1216–1219 (2008). 18830245 10.1038/nature07332

[b15] Takano T., Fuyama M., Namiki R. & Takahashi Y. Spin Squeezing of a Cold Atomic Ensemble with the Nuclear Spin of One-Half. Phys. Rev. Lett. 102, 033601 (2009). 19257352 10.1103/PhysRevLett.102.033601

[b16] Schleier-Smith M. H., Leroux I. D. & Vuletic V. States of an Ensemble of Two-Level Atoms with Reduced Quantum Uncertainty. Phys. Rev. Lett. 104, 073604 (2010). 20366883 10.1103/PhysRevLett.104.073604

[b17] Leroux I. D., Schleier-Smith M. H. & Vuletic V. Implementation of Cavity Squeezing of a Collective Atomic Spin. Phys. Rev. Lett. 104, 073602 (2010). 20366881 10.1103/PhysRevLett.104.073602

[b18] Lücke B. . Twin Matter Waves for Interferometry Beyond the Classical Limit. Science 334, 773–776 (2011). 21998255 10.1126/science.1208798

[b19] Kuzmich A., Mandel L. & Bigelow N. P. Generation of Spin Squeezing via Continuous Quantum Nondemolition Measurement. Phys. Rev. Lett. 85, 1594 (2000). 10970566 10.1103/PhysRevLett.85.1594

[b20] Gross C., Zibold T., Nicklas E., Estève J. & Oberthaler M. K. Nonlinear atom interferometer surpasses classical precision limit. Nature 464, 1165–1169 (2010). 20357767 10.1038/nature08919

[b21] Riedel M. F. . Atom-chip-based generation of entanglement for quantum metrology. Nature 464, 1170–1173 (2010). 20357765 10.1038/nature08988

[b22] Stamper-Kurn D. M. & Ueda M. Spinor Bose gases: Symmetries, magnetism, and quantum dynamics. Rev. Mod. Phys. 85, 1191–1244 (2013).

[b23] Hoang T. M. . Dynamic Stabilization of a Quantum Many-Body Spin System. Phys. Rev. Lett. 111, 090403 (2013). 24033006 10.1103/PhysRevLett.111.090403

[b24] Ho T.-L. Spinor Bose Condensates in Optical Traps. Phys. Rev. Lett. 81, 742–745 (1998).

[b25] Ohmi T. & Machida K. Bose-Einstein condensation with internal degrees of freedom in alkali atom gases. J. Phys. Soc. Jpn 67, 1822–1825 (1998).

[b26] Stenger J. . Spin domains in ground-state Bose-Einstein condensates. Nature 396, 345–348 (1999).

[b27] Chang M.-S. . Observation of Spinor Dynamics in Optically Trapped Rb^87^ Bose-Einstein Condensates. Phys. Rev. Lett. 92, 140403 (2004). 15089521 10.1103/PhysRevLett.92.140403

[b28] Huang Y., Sun Z. & Wang X. Atom-number fluctuation and macroscopic quantum entanglement in dipole spinor condensates. Phys. Rev. A 89, 043601 (2014).

[b29] Schmaljohann H. . Dynamics of *F* = 2 Spinor Bose-Einstein Condensates. Phys. Rev. Lett. 92, 040402 (2004). 14995355 10.1103/PhysRevLett.92.040402

[b30] Law C. K., Pu H. & Bigelow N. P. Quantum Spins Mixing in Spinor Bose-Einstein Condensates. Phys. Rev. Lett. 81, 5257–5261 (1998).

[b31] Yi S., You L. & Pu H. Quantum Phases of Dipolar Spinor Condensates. Phys. Rev. Lett. 93, 040403 (2004). 15323738 10.1103/PhysRevLett.93.040403

[b32] Huang Y., Zhang Y., Lü R., Wang X. & Yi S. Macroscopic quantum coherence in spinor condensates confined in an anisotropic potential. Phys. Rev. A 86, 043625 (2012).

[b33] Yi S. & Pu H. Magnetization, squeezing, and entanglement in dipolar spin-1 condensates. Phys. Rev. A 73, 023602 (2006).

[b34] Hald J., Sørensen J. L., Schori C. & Polzik E. S. Spin Squeezed Atoms: A Macroscopic Entangled Ensemble Created by Light. Phys. Rev. Lett. 83, 1319–1322 (1999).

[b35] Sau J. D., Leslie S. R., Cohen M. L. & Stamper-Kurn D. M. Spin squeezing of high-spin, spatially extended quantum fields. New. J. Phys. 12, 085011 (2010).

[b36] Kuzmich A., Mømer K. & Polzik E. S. Spin Squeezing in an Ensemble of Atoms Illuminated with Squeezed Light. Phys. Rev. Lett. 79, 4782–4785 (1997).

[b37] Sørensen J. L., Hald J. & Polzik E. S. Quantum Noise of an Atomic Spin Polarization Measurement. Phys. Rev. Lett. 80, 3487–3490 (1998).

[b38] Sewell R. J. . Magnetic Sensitivity Beyond the Projection Noise Limit by Spin Squeezing. Phys. Rev. Lett. 109, 253605 (2012). 23368463 10.1103/PhysRevLett.109.253605

[b39] Yukawa E., Ueda M. & Nemoto K. Classification of spin-nematic squeezing in spin-1 collective atomic systems. Phys. Rev. A 88, 033629 (2013).

[b40] Gerving C. S. . Non-equilibrium dynamics of an unstable quantum pendulum explored in a spin-1 Bose-Einstein condensate. Nat. Comm. 3, 1169 (2012). 10.1038/ncomms2179 23132019

[b41] Hamley C. D. . Spin-nematic squeezed vacuum in a quantum gas. Nat. Phys. 8, 305–308 (2012).

[b42] Pu H. & Meystre P. Creating macroscopic atomic Einstein-Podolsky-Rosen states from Bose-Einstein condensates. Phys. Rev. Lett. 85, 3987–3990 (2000). 11056606 10.1103/PhysRevLett.85.3987

[b43] Duan L.-M., Sønsen A., Cirac J. I. & Zoller P. Squeezing and entanglement of atomic beams. Phys. Rev. Lett. 85, 3991–3994 (2000). 11056607 10.1103/PhysRevLett.85.3991

[b44] Duan L.-M., Cirac J. I. & Zoller P. Quantum entanglement in spinor Bose-Einstein condensates. Phys. Rev. A 65, 033619 (2002).

[b45] Mias G. I., Cooper N. R. & Girvin S. M. Quantum noise, scaling, and domain formation in a spinor Bose-Einstein condensate. Phys. Rev. A 77, 023616 (2008).

[b46] Kajtoch D. & Emilia W. Spin squeezing in dipolar spinor condensates. Phys. Rev. A 93, 023627 (2016).

[b47] Armaitis J., Duine R. A. & Stoof H. T. C. Quantum Rotor Model for a Bose-Einstein Condensate of Dipolar Molecules. Phys. Rev. Lett. 111, 215301 (2013). 24313496 10.1103/PhysRevLett.111.215301

[b48] Baillie D., Bisset R. N. & Blakie P. B. Stability of a trapped dipolar quantum gas. Phys. Rev. A 91, 013613 (2015).

[b49] Nath R., Pedri P. & Santos L. Stability of Dark Solitons in Three Dimensional Dipolar Bose-Einstein Condensates. Phys. Rev. Lett. 101, 210402 (2008). 19113395 10.1103/PhysRevLett.101.210402

[b50] Wilson R. M. & Bohn J. L. Emergent structure in a dipolar Bose gas in a one-dimensional lattice. Phys. Rev. A 83, 023623 (2011).

[b51] Jin G.-R. & Kim S. W. Storage of Spin Squeezing in a Two-Component Bose-Einstein Condensate. Phys. Rev. Lett. 99, 170405 (2007). 17995306 10.1103/PhysRevLett.99.170405

[b52] Huang Y., Xiong H.-N., Sun Z. & Wang X. Generation and storage of spin-nematic squeezing in a spinor Bose-Einstein condensate. Phys. Rev. A 92, 023622 (2015).

